# UPBEAT Trial: a randomised feasibility evaluation of a digital system for UPper limB rEhabilitation After sTroke

**DOI:** 10.1186/s40814-025-01727-0

**Published:** 2025-11-07

**Authors:** Gianpaolo Fusari, Sarah Coleman, Jane Davies, Jeremy Dearling, Joanne Findlay, Mark Goddard, Nicola Ivins, Fiona Jones, Fiona Leggat, Rachel Lowe, Clare McCrudden, Richard McKinlay, Ebenezar Ndachi Effiang, Louise Penny, Rebecca Playle, Davina Richardson, Muhammad Riaz, Leila Shepherd, Tongtong Shi, Tomasz Szymanski, Ara Darzi

**Affiliations:** 1 Helix Centre, Institute of Global Health Innovation, Imperial College London, London, England UK; 2 Kingston and Richmond NHS Foundation Trust, London, England UK; 3 Centre for Trials Research, School of Medicine, Cardiff University, Cardiff, Wales UK; 4 PPIE representative, Norfolk, UK; 5 Discover-NOW, Imperial College Health Partners, London, UK; 6 Population Health Research Institute, City St George’s University of London, London, England UK; 7 Central London Community Healthcare NHS Trust, London, England UK; 8 Imperial College Healthcare NHS Trust, London, England UK

**Keywords:** Stroke rehabilitation, NHS stroke pathway, Self-directed therapy, Digital health intervention, Wearable technology, Feasibility trial, Process evaluation, Health economic assessment

## Abstract

**Background:**

Stroke rehabilitation is essential, yet many stroke survivors receive insufficient therapy, particularly for arm function. The 2023 UK National Stroke Guidelines recommend ongoing rehabilitation, but resource constraints limit therapy intensity. Digital interventions, such as OnTrack—a wearable system co-designed with stroke survivors, carers and therapists—offer a scalable self-management solution for rehabilitation. This study evaluates the feasibility of implementing OnTrack within NHS stroke services and informs a future RCT.

**Methods:**

A two-arm, parallel-group randomised feasibility trial conducted across three NHS sites. Stroke survivors (*n* = 30) with arm weakness within 12 months post-stroke were randomised 1:1 to receive either OnTrack plus usual care or usual care alone. The primary outcome was the recruitment rate. Secondary feasibility outcomes included retention, adherence, and intervention fidelity. Secondary clinical measures assessed arm function (MAL-14), self-efficacy (SSEQ), quality of life (EQ-5D-5L), and usability (SUS). A process evaluation explored intervention fidelity and implementation experiences. A preliminary economic evaluation examined data collection feasibility using the Client Service Receipt Inventory (CSRI) and a per-patient costing of the OnTrack intervention.

**Results:**

Thirty participants out of a target of 42 (71.4%) were randomised, with 24/30 (80%) retained at 12 weeks. Intervention adherence was high with participants engaging with OnTrack an average of 6.3 days per week (90.7%). Fidelity of intervention delivery was modest (53.9%), highlighting areas for therapist training improvements. Trends suggested improvements in arm function and self-efficacy across groups. The preliminary economic evaluation confirmed the feasibility of data collection using CSRI, though limitations in accuracy and completeness were identified. A per-patient costing of the OnTrack intervention was achieved. Apparent cost differences between groups, identified through a preliminary cost-consequence analysis, were influenced by an unbalanced distribution of reported service use and should be interpreted with caution.

**Conclusions:**

UPBEAT demonstrated the feasibility of delivering OnTrack in NHS stroke services, with strong retention and adherence. Findings support the potential for a future full-scale RCT, with refinements needed in recruitment procedures, therapist training, and economic data collection methods. Digital self-management tools like OnTrack may offer a scalable approach to increasing rehabilitation intensity, addressing gaps in stroke recovery pathways.

**Trial registration:**

UPBEAT Feasibility Trial, IRAS: 323576, CPMS: 59352. Approved by London Surrey REC on 14/12/2023.

## Roles and responsibilities

Imperial College London acted as the Sponsor for this trial; please refer to the Sponsor Details section in the Supplementary Information for further details.

The sites are Charing Cross Hospital, London; Community Neuro-Rehabilitation Team for Richmond, London; Community Neuro-Rehabilitation Team for Hammersmith & Fulham, Kensington, Chelsea & Westminster, London.

Patient and Public Involvement and Engagement (PPIE) activities and input were completed with support from the Helix Centre’s Engagement Lead.

## Key messages regarding feasibility

1. What uncertainties existed regarding feasibility?
  This was the first time the OnTrack intervention was delivered by NHS stroke therapy teams, as such, it was uncertain whether it could be successfully integrated into NHS stroke rehabilitation services. Additionally, the acceptability of trial procedures and feasibility of recruitment, retention, intervention adherence, and fidelity of delivery required evaluation. Furthermore the feasibility of health resource use data collection from patients and therapists for the health economic analysis needed appraisal.
2. What are the key feasibility findings?
  The trial demonstrated that recruitment and retention were achievable within NHS stroke services. Recruitment rates were consistent with previous similar studies, however an extension of the recruitment period was needed to achieve this. Intervention adherence was high, with participants engaging with OnTrack on 6.3 days per week. Fidelity of intervention delivery was moderate, highlighting a need for improved therapist training. The feasibility of collecting health economic data was established, and a detailed per-patient costing of the OnTrack intervention was conducted. Refinements are needed to improve the accuracy and completeness of health resource use data.
3. What are the implications of the feasibility findings for the design of the main study?
  The findings support the feasibility of a full-scale RCT, with refinements needed to optimise recruitment strategies, therapist training and data collection methods. Future studies should consider expanding eligibility criteria to patients receiving arm rehabilitation regardless of time since stroke onset to improve recruitment. Incorporating objective measures of arm activity in the control group may also strengthen outcome comparisons. Improving economic data collection methods will support more robust cost-effectiveness analyses.

## Introduction

### Background

Each year, over 100,000 people in the UK experience a stroke [1]. Whilst advances in stroke prevention and acute care have improved survival rates, more people are living with long-term disabilities that require rehabilitation [2]. Stroke-related costs are expected to rise significantly, from £3.4 billion in 2015 to £10.2 billion by 2035 [3]. Despite the potential for recovery [4], fewer than half of stroke survivors regain basic arm function within a year, which greatly limits their independence and quality of life [5–7]. Arm activity levels post-stroke are closely linked to broader participation and activity in daily life [8]. This underscores the importance of effective rehabilitation to support arm recovery after stroke.

The 2023 UK National Stroke Guidelines highlight the potential for rehabilitation to benefit all stroke survivors, emphasising that improvement is contingent on receiving an ‘effective dose’ of therapy [9]. However, with increasing stroke prevalence, many patients have limited access to sufficient therapy [10]. Opportunities to engage in self-directed or group activities that promote recovery are also scarce [11]. There is an urgent need for scalable solutions to support frequent, purposeful arm activities that can drive functional recovery [12].

The recent NHS review conducted by Lord Darzi further underscores this issue, revealing that over 1 million people are currently waiting for community services, with more than 50,000 experiencing delays of over a year [13]. Such delays exacerbate the challenge of delivering timely and effective rehabilitation.

### Rationale for the study

Innovative approaches, including robotic-assisted therapy [14] and virtual reality [15, 16], show promise in improving arm function. However, these technologies often face barriers to integration into routine clinical practice [17], with some, such as robotic therapy, recommended only in research settings [9]. Surveys of UK upper limb rehabilitation practices reveal inconsistent adoption of evidence-based treatments and highlight the need for solutions that are both effective and feasible within current clinical workflows [18].

OnTrack is a digital intervention developed in collaboration with stroke survivors, carers, and therapists to increase the practice of arm activities outside of formal therapy sessions. The platform combines wearable technology, real-time activity feedback, and self-management coaching to enhance rehabilitation. An initial non-randomised study demonstrated promising outcomes, including increased arm activity and performance, improved confidence in activity engagement, and high levels of usability and acceptability [19, 20]. Building on these findings, this trial aimed to evaluate the feasibility and acceptability of trial procedures and intervention delivery to patients and clinicians of OnTrack through a randomised controlled design.

### Objectives

The primary aim of this trial was to assess the feasibility of implementing OnTrack within NHS stroke services across the treatment pathway and to determine its potential for progression to a definitive randomised controlled trial (RCT). Specific objectives included evaluating:

- Trial feasibility, focusing on recruitment, retention, and data collection processes.
- Intervention feasibility, including fidelity of delivery and adherence.

Secondary objectives were to gather preliminary trends of clinical outcomes and conduct a preliminary cost consequence analysis to estimate the economic impact of OnTrack alongside usual care compared to usual care alone. By addressing these objectives, the trial sought to inform future large-scale evaluations and guide the integration of digital innovations into routine stroke rehabilitation pathways.

## Methods

### Study design

We conducted a two-arm, parallel-group randomised feasibility trial comparing the OnTrack Rehab intervention plus usual care with usual care alone with an allocation ratio of 1:1. A nested process evaluation and economic evaluation were performed in parallel with the trial.

The study followed a pre-registered protocol (ISRCTN: 87799346; CPMS: 59352) developed following SPIRIT reporting guidelines [21, 22]. All amendments to the protocol after trial commencement, such as adjustments to eligibility criteria or procedures, were documented with justifications and approved by the Health Research Authority, London Surrey Research Ethics Committee.

Overall progression of the study was monitored through regular meetings with the Trial Monitoring Group (TMG) and Trial Steering Committee (TSC) at which data on recruitment, protocol adherence, follow-up, and adverse events were reported and discussed in depth.

### Participants and recruitment

The study involved the recruitment of patients and therapists.

#### Patient participants

Stroke survivors aged ≥ 18 years with arm weakness who were within 12 months post-stroke were eligible to participate. Arm weakness was defined pragmatically by the recruiting therapist using clinical judgement, based on standard assessments and observations. Patient participants needed to be receiving rehabilitation care from one of the recruitment sites, be English speakers with a reliable communication method (e.g., verbal or non-verbal), and be able to provide informed consent. Exclusion criteria included unstable medical conditions, severe pain or oedema in the affected arm, hemianopia, or other conditions deemed unsuitable by the Principal Investigator at the site.

Originally, the inclusion criteria specified a 6-month post-stroke maximum timeframe; however, after an analysis of the initial 3 months of screening data, the team identified that a number of potential participants had been excluded from participation on account of exceeding this 6-month window. Extending the inclusion criterion allowed the study to increase the pool of eligible participants and assess how this could affect overall trial recruitment rates.

#### Therapist participants

Qualified individuals responsible for providing rehabilitation treatment for stroke patients within the designated recruitment sites were recruited (e.g., physiotherapists, occupational therapists, speech and language therapists, and rehabilitation assistants).

Recruitment occurred across NHS stroke services in London, including Imperial College NHS Trust, Central London Community Healthcare NHS Trust and Kingston and Richmond NHS Foundation Trust (formerly Hounslow and Richmond Community Healthcare NHS Trust). Participants were identified by therapists at the participating sites and provided with detailed study information. Written informed consent was obtained before randomisation.

Data collection was conducted in both hospital and community settings through face to face and telephone sessions primarily using digital forms in the Research Electronic Data Capture platform (REDCap) [23, 24]. Paper versions of the forms were available for those requiring them.

### Intervention

Within 14 days of randomisation, patients randomised to the intervention arm received usual care plus the OnTrack Rehab intervention [25] combining arm activity tracking using a smartwatch and a programme of self-management coaching based on principles developed by Bridges Self-Management [26]. Participants were asked to track their activity on a daily basis during waking hours. Participants were coached in self-management by their treating therapist during the intervention onboarding session and follow-up sessions. The Bridges + OnTrack training provided therapists with structured guidance for these sessions, including prompts for supporting behaviour change techniques. The OnTrack intervention did not involve the prescription of specific exercises outside of those already provided as part of usual care. Instead, the intervention aimed to support participants in developing strategies to increase use of their impaired arm through self-directed practice of functional, everyday activities, real-time feedback, and personalised coaching. The intervention was delivered over 12 weeks as advocated by habit-formation studies in the literature [27, 28].

The control group received usual care as per NHS guidelines [9], which typically included therapy provided by multidisciplinary teams in acute and community settings.

Usual care was not standardised and reflected the service delivered at each site, including inpatient, community, or Early Supported Discharge services. The nature and intensity of therapy varied by site, therapist availability, and individual patient needs.

### Therapist training

Before the start of recruitment, therapists delivering the intervention received a 6-h training package divided into 4 remote sessions (MS Teams) covering the use of the OnTrack software applications and the Bridges self-management coaching approach. On-going support was provided to therapists on an ad hoc basis, and via a study digital repository containing crib-sheets and guidance on the coaching language to use, as well as troubleshooting guides for OnTrack. Therapists were part of usual care teams and, depending on patient allocation, delivered both usual care and the intervention.

### Outcomes

The trial’s primary outcome was the *recruitment rate* (percentage of eligible patients who consent and are randomised). Secondary feasibility outcomes included *retention rates* (percentage of randomised participants retained in the study and complete outcome measures at 6 and 12 weeks follow-ups), *intervention adherence* (percentage of days the system is actively used by participants), and fidelity of *intervention delivery* (assessed during process evaluation observations using a predefined checklist developed for a prior feasibility trial and adapted for this trial [19, 20]).

In addition, secondary clinical outcomes were collected at baseline (in person), 6 and 12 weeks (in person or remotely) using REDCap. These included arm function and quality of movement (MAL-14) [29], self-efficacy (SSEQ) [30] (N.B. 4 additional items relating to arm activity were included for this study on the basis of PPI engagement activities), quality of life (EQ-5D-5L) [31], and stroke-related disability/dependence through the Modified Rankin Scale (mRS) [32]. The System Usability Scale (SUS) [33] was used only with participants in the intervention arm to assess the usability of the OnTrack user interface and experience. Measures of arm activity (average minutes of activity, average target minutes, days target reached, days recording activity) were measured for those participants in the intervention group using the OnTrack system.

Progression criteria (Table 1) were determined by the Trial Management Group with input from the Trial Steering Committee based on the primary feasibility outcome (recruitment rate), and secondary outcomes (retention, adherence, fidelity, and delivery metrics) to inform the progression to a definitive phase III RCT.

**Table 1:** Progression criteria

Measure	Green: Proceed to phase III RCT as planned	Amber: Proceed to phase III RCT with changes	Red: Do not proceed to phase III RCT
**1. Recruitment** % eligible who consent and are randomised	100%	> 30% & < 100%	< 30%
**2. Retention** % retained in study and complete measures at 6/12 weeks follow-up	100%	> 50% & < 100%	< 50%
**3. Adherence** % adhere to intervention	Participants activated the OnTrack system on a minimum of 75% of days	Participants activated the OnTrack system between 75 and 50% of days	Participants activated the OnTrack system less than 50% of days
**4. Fidelity of intervention delivery** Fidelity of OnTrack intervention delivery	An average of at least 75% on fidelity scores	An average between 50–74% on fidelity scores	An average of less than 50% on fidelity scores

### Health economic evaluation

A feasibility health economic analysis was conducted from the perspective of the NHS and Personal Social Services (PSS). The aim was to assess the feasibility of collecting resource use data and per-patient costing the OnTrack intervention within the context of the UPBEAT Trial. Following guidance from the Evidence Standard Framework for Digital Health Technologies [34], a preliminary Cost Consequence Analysis (CCA) framework was used. The analysis did not seek to determine cost-effectiveness but instead focus on data completeness, costing methodology, and areas requiring refinement ahead of a full trial.

The Client Services Receipt Inventory (CSRI) [35] was used to obtain data at 6 weeks and 12 weeks on health resource use related to patient arm impairment for both the intervention and control cohorts. The CSRI also covered out of pocket expenses, these are reported separately as they fall outside of NHS and Personal Social Services (PSS). Data on the number of days that participants spent in inpatient care (Hyper-Acute Stroke Units [HASU] and Acute Stroke Units [ASU]) and the number of outpatient sessions delivered through Early Supported Discharge (ESD) and Community Neurological Rehabilitation Teams (CNRT) were recorded by treating therapists. These data were mapped to NHS unit costs to estimate the cost of usual care for both trial arms. Health resource use was mapped to publicly available cost data, such as unit costs of health and social care data provided by Personal Social Services Research Unit PSSRU [36], obtained via a targeted literature review. The PSSRU conducts high quality and validated research to inform and influence social and healthcare policy, practice and theory nationally and internationally.

The cost of delivering OnTrack during the feasibility trial was estimated using a per-patient costing approach. Per-patient costing involved identifying individual resource components used during intervention delivery and applying corresponding unit costs to produce a detailed estimate of overall cost [37]. All resource components were identified and valued using unit costs from the PSSRU and internal project records. Components included the provision of devices (smartwatch and smartphone), technical support, therapist training, therapist time spent on follow-up calls, and time spent consulting the OnTrack Tools platform.

EQ-5D-5L data were collected at baseline, 6 weeks and 12 weeks, and converted into EQ-5D-3L utility scores using the Van Hout crosswalk method [38], in line with the NICE position statement [39]. Utility data were not used to calculate quality-adjusted life years (QALYs) in this feasibility study but were assessed for completeness and suitability for future economic modelling.

### Process evaluation

An independent process evaluation was undertaken by FJ and FL. This aimed to explore therapists’ and patients’ experiences of using OnTrack and understand the factors influencing its delivery as an adjunct to usual care. Interviews at the end of participation were carried out over phone or face to face with intervention and usual care participants, and online focus groups were conducted with therapists. A subset of intervention sessions were observed to assess fidelity, using a predefined checklist of 10 key coaching skills. Qualitative data were analysed using a combination of inductive and deductive analysis, informed by the Consolidated Framework for Implementation Research (CFIR v2) [40]. The results of the process evaluation will be reported fully in a separate publication.

### Follow-up

Participants from both study groups were reassessed by the research team at the 6- and 12-week time points. Patient participants were contacted by telephone to complete a follow-up of the baseline questionnaires plus the CSRI. In addition, individuals in the intervention arm completed the SUS questionnaire.

At the 3- and 6-month timepoints, therapists delivering the intervention were asked to complete the SUS.

### Sample size

The target sample size of 42 participants (21 per arm) was based on feasibility considerations rather than statistical power, aligning with the trial’s primary aim of assessing feasibility. Based on this sample size, and screening a pool of 129 eligible participants across three sites, the two-sided 95% confidence interval for the proportion of participants randomised would extend ± 8.1 percentage points from the observed proportion, assuming an expected consent rate of 32.6% [41].

### Randomisation

Randomisation took place a maximum of 7 days after participants provided consent and completed baseline assessments. These assessments included collecting demographic and health-related information, as well as administering the MAL-14, SSEQ, EQ-5D-5L, and mRS questionnaires.

### Sequence generation, allocation, and implementation

The trial statistician generated the random allocation sequence using a computerised randomisation programme in STATA [42] and implemented within REDCap. Random permuted block randomisation, stratified by site, was employed to ensure each participant had an equal chance of assignment to either group. Block sizes of 2, 4, and 6 were used to reduce predictability of randomisation allocations, with each block containing an equal number of intervention group allocations. The order of treatments within each block was randomly permuted, and a random number sequence selected the block arrangement, determining the allocation order for each set of participants, with the process repeated for subsequent participants. The allocation sequence was concealed within the REDCap system, preventing any access until participants had been enrolled and baseline assessments were completed. Following this step, REDCap automatically assigned participants in a 1:1 ratio to either the intervention or control groups.

### Blinding

Participants and therapists were not blinded due to the nature of the intervention. Only the trial statistician was kept blinded to the participant allocation to the randomisation arms.

### Statistical analysis

The primary analysis summarised feasibility metrics, such as recruitment, retention, adherence, and fidelity. Feasibility outcomes were summarised descriptively using counts, proportions, means, and standard deviations.

*Recruitment* rates were calculated as a proportion of all screened patients who met the eligibility criteria that were consented and randomised into the trial.

*Retention* rates were calculated as a percentage of all recruited participants who were retained in the study and completed follow-up measures at 6 and 12 weeks.

*Intervention adherence* was calculated as the average percentage of days that the OnTrack system was actively used by patient participants; in this context, usage is defined as participants making a conscious decision to start an activity recording session each day of the intervention period. Intervention usage data was captured at each time point participants engaged with the intervention and tabulated on a weekly basis.

*Intervention delivery fidelity* was evaluated based on ratings assigned to ten predefined key coaching skills from a dedicated fidelity checklist. Observers assessed each skill using a 4-point Likert scale ranging from 0 to 3, where 0 indicated the skill was not observed, 1 indicated it was rarely observed, 2 indicated it was sometimes observed, and 3 indicated it was mostly observed. The ratings for all ten skills were summed to produce an overall session score, with a maximum possible score of 30. This overall score was then converted into a percentage (e.g., 30 equating to 100%), with higher percentages indicating greater fidelity in intervention delivery by the therapists. Fidelity outcomes will be reported in full in a separate publication.

Clinical outcome measures (EQ-5D-5L [43, 44], SSEQ [45, 46], MAL14 [47], mRS [48]) were scored as per measure instructions at each time point and tabulated per arm and overall.

Data completeness was monitored for all outcomes at each time point. Statistical analyses were carried out in STATA 18 [42].

## Results

### Participant flow

#### Participant characteristics

Thirty patients consented to participate and were randomly assigned to the two treatment arms—15 to the intervention group and 15 to the control group. Table 2 shows the study population characteristics. The target follow-up period was 12 weeks or roughly 84 days; however, the median follow-up time for all participants was 92 days (range 22–173 days). In some cases, assessments were delayed due to logistical factors such as scheduling difficulties, whilst in the most extreme cases, extended follow-up was related to adverse events leading to hospitalisation or temporary suspension from trial activities.

**Table 2:** Participant characteristics at baseline

Characteristic	Intervention (*n* = 15)	Usual care (*n* = 15)	Total (*n* = 30)
Age (median (IQR, range))	68.0 (58.0–74.0, 49.0–81.0)	66.0 (63.0–76.0, 56.0–87.0)	68.0 (63.0–75.0, 49.0–87.0)
**Sex (n (%))**			
Male	7 (46.7)	7 (46.7)	14 (46.7)
Female	8 (53.3)	8 (53.3)	16 (53.3)
**Ethnicity (n (%))**			
Asian or Asian British	2 (13.3)	2 (13.3)	4 (13.3)
Black, Black British, Caribbean or African	2 (13.3)	3 (20.0)	5 (16.7)
Mixed or multiple ethnic groups	0 (0.0)	1 (6.7)	1 (3.3)
White	10 (66.7)	9 (60.0)	19 (63.3)
Other ethnic group	1 (6.7)	0 (0.0)	1 (3.3)
**Smartphone user (*n* (%))**			
No	1 (6.7)	0 (0.0)	1 (3.3)
Yes	14 (93.3)	15 (100.0)	29 (96.7)
**Wi-fi at home (*n* (%))**			
No	0 (0.0)	0 (0.0)	0 (0.0)
Yes	15 (100.0)	15 (100.0)	30 (100.0)
**Participant enrolled in other studies (*n* (%))**			
No	13 (86.7)	11 (73.3)	24 (80.0)
Yes	2 (13.3)	4 (26.7)	6 (20.0)
**Impaired arm (*n* (%))**			
Left	8 (53.3)	6 (40.0)	14 (46.7)
Right	7 (46.7)	9 (60.0)	16 (53.3)
**Dominant arm (*n* (%))**			
Left	1 (6.7)	3 (20.0)	4 (13.3)
Right	14 (93.3)	12 (80.0)	26 (86.7)
**Dominant arm impaired (*n* (%))**			
No	9 (60.0)	7 (46.7)	16 (53.3)
Yes	6 (40.0)	8 (53.3)	14 (46.7)
**First or recurrent stroke (*n* (%))**			
First	14 (93.3)	10 (66.7)	24 (80.0)
Recurrent	1 (6.7)	5 (33.3)	6 (20.0)
**Type of stroke (*n* (%))**			
Ischemic	13 (86.7)	12 (80.0)	25 (83.3)
Hemorrhagic	2 (13.3)	3 (20.0)	5 (16.7)
**Subtype of ischemic stroke (*n* (%))**			
Large artery atherosclerosis	3 (23.1)	2 (16.7)	5 (20.0)
Cardioembolism	1 (7.7)	4 (33.3)	5 (20.0)
Small-vessel occlusion	2 (15.4)	2 (16.7)	4 (16.0)
Undetermined	4 (30.8)	2 (16.7)	6 (24.4)
Other	3 (23.1)	2 (16.7)	5 (20.0)
**Other subtype of ischaemic stroke (*n* (%))**			
Atrial fibrillation	0 (0.0)	1 (50.0)	1 (20.0)
Corona radiata lacunar infarct	1 (33.3)	0 (0.0)	1 (20.0)
L MCA infarct	1 (33.3)	0 (0.0)	1 (20.0)
Pontine	1 (33.3)	0 (0.0)	1 (20.0)
Low density in the left lentiform nucleus and posterior limb of the internal capsule suggestive of an evolving infarct	0 (0.0)	1 (50.0)	1 (20.0)
**Subtype of hemorrhagic stroke (*n* (%))**			
Intracerebral	2 (100.0)	3 (100.0)	5 (100.0)
**Comorbidities (*n* (%))**			
No	3 (20.0)	4 (26.7)	7 (23.3)
Yes	12 (80.0)	11 (73.3)	23 (76.7)
**Smoking history (*n* (%))**			
Current smoker	2 (13.3)	1 (6.7)	3 (10.0)
Quit within last 6 months	1 (6.7)	0 (0.0)	1 (3.3)
Quit more than 6 months ago	4 (26.7)	2 (13.3)	6 (20.0)
Never smoked	8 (53.3)	12 (80.0)	20 (66.7)
**Recruiting site**			
Acute	5	8	13
Community	10	7	17

In addition, 37 therapists were recruited to deliver the intervention to patient participants.

### Feasibility outcomes

A summary of the main feasibility outcomes can be seen on Table 3.

**Table 3:** Summary of feasibility outcomes

**Outcome**	**Result**	**95% CI (lower, upper)**
Recruitment rate (*n*/*N* (%))	30/77 (39.0)	28.1, 50.8
Retention rate (*n*/*N* (%))	24/30 (80.0)	61.4, 92.3
Adherence rate (mean % (SD))^a^	90.7 (0.83)	90.2, 91.2
Fidelity of intervention delivery (mean % (SD))^b^	53.9 (13.2)	45.5, 62.3

^a^Adherence measured as mean percentage of days the system was used over a 84-day period

^b^Fidelity measured as mean percentage of key intervention components delivered (max score = 30)

### Recruitment

**Fig. 1:**
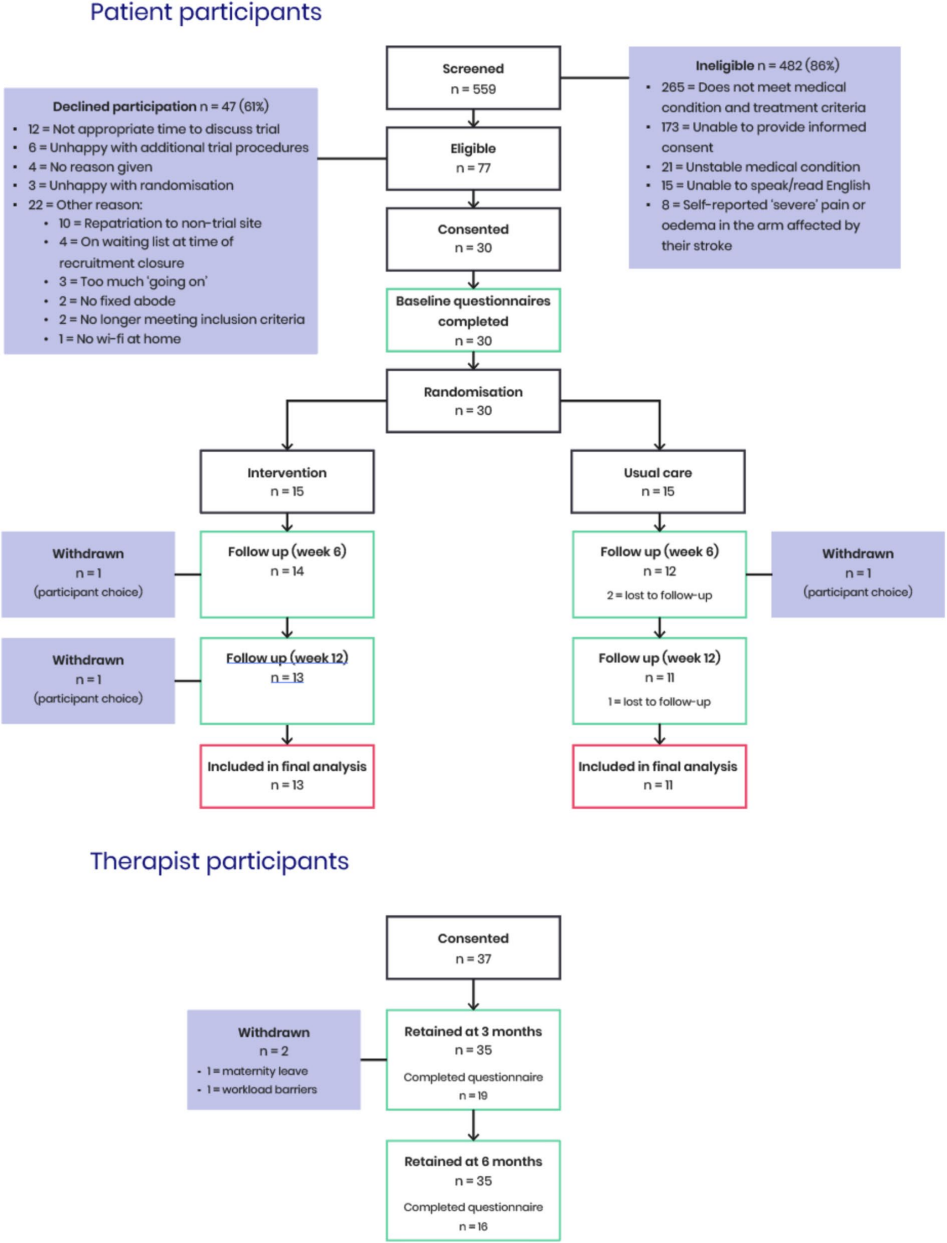
UPBEAT Trial CONSORT diagram

The study was open to recruitment between 07/02/2024 and 31/07/2024, a total of 38 weeks after being extended for 8 weeks due to low recruitment rates. Screening of 559 patients across sites found 77 (13.8%) to be eligible and were invited to participate in the study. Reasons for ineligibility can be seen in Fig. 1. Thirty patients (39% of those eligible (95% CI 28.1–50.8%)) accepted participation and were randomised, reaching an amber score in our predefined progression criteria (Table 1). Forty-seven declined participation or were excluded after being invited, reasons can be seen in Fig. 1.

All participants were recruited from one acute and two community NHS Trusts in London providing stroke rehabilitation services. Thirty participants were recruited out of the 42 the study aimed to recruit (71.4% of the target), with a recruitment rate of 0.79 participants per week, or 3.33 per month. The recruitment rate was slightly lower than the average of 1.5 participants per month per site seen in similar stroke rehabilitation trials worldwide but in line with other European studies (1 participant per month per site on average) [41]. Recruitment and retention data is shown in Table 4.

**Table 4:** Recruitment and retention outcomes

Trial feasibility outcomes (*n*/*N* (%))	Intervention	Usual care	Total
Recruitment	15/21 (71.4)	15/21 (71.4)	30/42 (71.4)
Withdrawals	2/15 (13.3)	1/15 (6.6)	3/30 (10)
Follow-up (week 6)	14/15 (93.3)	12/15 (80.0)	26/30 (86.6)
Follow-up (week 12)	13/15 (86.6)	11/15 (73.3)	24/30 (80.0)

### Retention

Thirty participants completed baseline assessments and were subsequently randomised to intervention or usual care. Twenty four participants (80% of those recruited (95% CI 61.4–92.3%)) completed follow-up assessments at 6 and 12 weeks, showing strong retention and placing an amber status in our progression criteria (Table 1). Three participants withdrew from the study (2 in the intervention and 1 in the control group), reasons for withdrawal were strictly due to participant choice and mainly related to feelings of having ‘too much on’ at that particular point in time as a result of their stroke. Three participants were lost to follow-up, all of whom were in the control group. As reported in previous studies [49], attrition tends to be higher in usual care groups, possibly to reduced engagement or perceived lack of benefit, which warrants further consideration for future trials.

### Intervention adherence

**Fig. 2:**
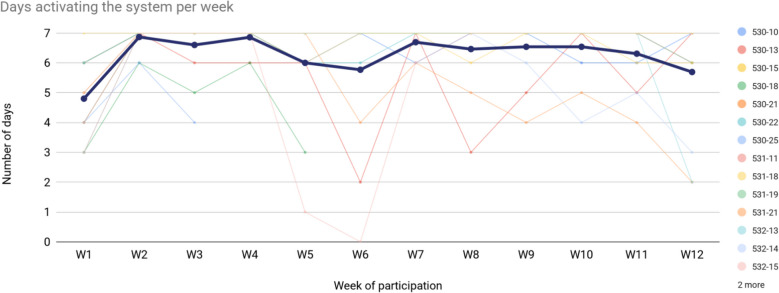
Number of days participants activated the system per week

Participants in the intervention group were provided with the OnTrack system. Adherence to the intervention was measured based on the number of days a participant activated the system by recording their arm activity. Thirteen out of 15 participants recruited to the intervention arm completed the 12-week intervention. Figure 2 shows participants completing the intervention period engaging with the OnTrack system an average of 6.3 days per week. Engagement was defined as wearing the smartwatch, launching the OnTrack app, and actively pressing ‘start’ to begin tracking arm activity. This corresponded to an excellent adherence of 90.7% (95% CI 90.2–91.2%) and a green status in our progression criteria (Table 1).

### Fidelity of intervention delivery

Fidelity of intervention delivery was assessed in a subset of intervention sessions using a predefined fidelity checklist reflecting the 10 key coaching skills observed. Outcomes reached an amber status (53.9% 95% CI 45.5–62.3%) in our progression criteria (Table 1), full results of the fidelity assessment are reported as part of a separate publication detailing all components including participant interviews, and therapy focus groups which formed part of the nested mixed methods process evaluation.

### Clinical outcomes

Clinical outcomes were collected as part of feasibility for data completeness and to assess initial data on variability and effects direction as a secondary outcome (Table 5). Participants in both groups were assessed at baseline, 6 and 12 weeks, covering quality of life (EQ-5D-5L), self-efficacy (SSEQ), motor activity (MAL-14), and functional outcomes (mRS). Intervention participants also completed usability assessments (SUS) at 6 and 12 weeks. As the primary and secondary outcomes focused on feasibility, between-group differences should not be over-interpreted, though effects were consistently positive. Data completion rates were high at baseline and follow-up.

**Table 5:** Outcome measure scores at baseline 6- and 12-week follow-up

Outcome	Effect direction	Baseline		6-weeks		12-weeks	
		**Intervention** *n*, Median [IQR]	**Usual care** *n*, Median [IQR]	**Intervention** *n*, Median [IQR]	**Usual care** *n*, Median [IQR]	**Intervention** *n*, Median [IQR]	**Usual care** *n*, Median [IQR]
EQ-5D-5L (index)	Higher scores indicate better health-related quality of life	15, 0.416 [0.050 to 0.536]	15, 0.502 [−0.042 to 0.672]	14, 0.543 [0.374 to 0.590]	12, 0.529 [0.340 to 0.700]	13, 0.636 [0.517 to 0.706]	11, 0.650 [0.441 to 0.822]
EQ-5D-5L (VAS)	Higher scores indicate better self-rated health	15, 50.0 [40.0 to 70.0]	15, 50.0 [10.0 to 70.0]	14, 60.0 [50.0 to 70.0]	12, 80.0 [55.5 to 92.5]	13, 52.0 [45.0 to 62.0]	11, 70.0 [50.0 to 80.0]
SSEQ	Higher scores indicate greater stroke self-efficacy	15, 16.0 [9.0 to 20.0]	15, 21.0 [11.0 to 31.0]	14, 25.0 [17.0 to 30.0]	12, 26.5 [22.0 to 42.5]	13, 31.0 [24.0 to 33.0]	11, 32.0 [21.0 to 48.0]
MAL-14 (how much)	Higher scores indicate more use of the affected limb	15, 0.571 [0.000 to 1.769]	15, 0.571 [0.071 to 1.786]	14, 2.110 [0.893 to 2.750]	12, 1.821 [0.536 to 3.500]	13, 1.964 [1.357 to 3.214]	11, 1.714 [0.143 to 4.929]
MAL-14 (how well)	Higher scores indicate better quality of movement in the affected limb	15, 0.571 [0.000 to 1.429]	15, 0.643 [0.143 to 1.385]	14, 2.339 [0.821 to 3.107]	12, 1.949 [0.464 to 3.125]	13, 2.000 [1.429 to 3.214]	11, 1.857 [0.429 to 4.036]
mRS	Lower scores indicate less severe disability	15, 3 [3 to 4]	15, 3 [2 to 4]	13, 3 [3 to 3]	12, 3 [1 to 4]	13, 2 [2 to 3]	11, 2 [1 to 3]
SUS^a^	Higher scores indicate greater perceived usability			14, 75.0 [70.0 to 80.0]		13, 77.5 [72.5 to 77.5]	

^a^SUS was only performed on participants in the intervention arm at 6- and 12-week follow-up

At baseline, median scores for most outcome measures were comparable between the intervention and control groups, reflecting similar initial conditions. Over the follow-up period, trends were observed. Overall, the intervention does not appear to be having any negative effects and has shown to be safe for the next stage of research in a larger trial.

Both arms showed improvement on the EQ-5D-5L (index), with higher scores at 12 weeks, suggesting an increase in health-related quality of life. On EQ-5D-5L (VAS), scores improved for both groups, indicating better self-rated health at follow-ups.

Stroke self-efficacy as shown by the SSEQ improved in both arms, with higher median scores at each time point. Both the ‘how much’ and ‘how well’ scores on the MAL improved, reflecting increased arm and hand usage and quality of movement of the affected limb. On the Modified Rankin Scale (mRS), disability severity decreased in both groups, with lower scores at 12 weeks. The proportion of participants with ‘slight’ or ‘moderate’ disability increased.

The median (IQR) score for the SUS at 12 weeks in the intervention group was 77.5 (72.5 to 77.5). This indicates that patients reported a good to excellent user experience with the intervention, as scores above 68 generally reflect above-average usability [50].

### Intervention related data

**Fig. 3:**
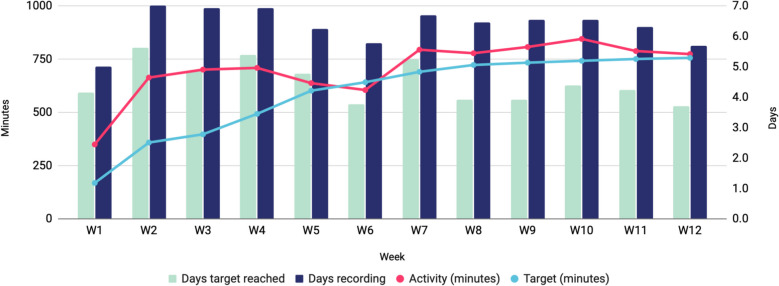
Arm activity trends as recorded by the OnTrack system

Participants in the intervention arm used OnTrack to record arm activity over 12 weeks. In total, we obtained complete 12-week activity data for 13 participants. Figure 3 shows key metrics: average days of activity recording, days targets were met and weekly activity minutes.

Participants showed strong engagement, recording activity an average of 6.3 days per week over 12 weeks, highlighting OnTrack’s feasibility for sustained rehabilitation. Activity targets were consistently exceeded, with achieved minutes surpassing targets every week with the exception of week 6. Participants met activity targets on average 4.5 days per week, reinforcing OnTrack’s support for goal-oriented therapy. These results indicate OnTrack’s potential to sustain arm activity, promote goal achievement and enhance therapy intensity—crucial for post-stroke recovery.

### Therapist usability

Usability of the clinician-facing application (OnTrack Tools) was measured using the SUS at the 3- and 6-month point from the start of recruitment (Table 6). Out of 37 therapists recruited, only 16 went on to use OnTrack Tools for delivering the intervention, a reflection on the number of participants in the intervention arm. At the 3-month point, 19 responses of the SUS were collected, these came from 9 participants who had used the system with patients and 10 who had not had the opportunity at that point. Three months later, at the 6-month point, 16 responses were received, 11 from therapists involved with participants and 5 from those who had not had this opportunity.

**Table 6:** Therapist usability scores at 3- and 6-month follow-up

Outcome	Effect direction	3-months		6-months	
		**All respondents** *n*, Median [IQR]	**Active users** *n*, Median [IQR]	**All respondents** *n*, Median [IQR]	**Active users** *n*, Median [IQR]
**SUS**	Higher scores indicate greater perceived usability	19, 60.0 [47.5 to 70.0]	9, 65.0 [55.0 to 72.5]	16, 66.25 [60.0 to 75.0]	11, 65.0 [61.3 to 71.3]

The median (IQR) score for the SUS at 3 months for all respondents was 60.0 (47.5 to 70.0) and 65.0 (55.0 to 72.5) for active users. There was a slight increase for all respondents at 6 months with a median score of 66.25 (60.0 to 75.0) and no change for active users (65.0 (61.3 to 71.3)). Scores show that therapists reported an ok user experience, just under the 50th percentile benchmark (score of 68) for SUS scores [50].

### Data completeness

Data completion rates for forms and questionnaires at different time points were high, with 100% completeness at baseline and 80% at 12 weeks demonstrating the feasibility of data collection (Table 7).

**Table 7:** Completion rates for questionnaires and forms

Form or questionnaire	Completion (*n*/*N*)	%
**Patient forms**		
Patient introduction	30/30	100
Participant registration	30/30	100
Eligibility	30/30	100
Patient consent online	20/30	66.7
Patient consent paper	10/30	33.3
Patient consent telephone	0/30	0
Randomisation form	30/30	100
Patient start	30/30	100
Patient questionnaires		
EQ-5D-5L (baseline)	30/30	100
SSEQ (baseline)	30/30	100
MAL-14 (baseline)	30/30	100
mRS (baseline)	30/30	100
EQ-5D-5L (week 6)	26/30	86.7
SSEQ (week 6)	26/30	86.7
MAL-14 (week 6)	26/30	86.7
mRS (week 6)	25/30	83.3
CSRI (week 6)	26/30	86.7
SUS (week 6)*	14/15	93.3
EQ-5D-5L (week 12)	24/30	80
SSEQ (week 12)	24/30	80
MAL-14 (week 12)	24/30	80
mRS (week 12)	24/30	80
CSRI (week 12)	24/30	80
SUS (week 12)*	13/15	86.7
**Therapist forms**		
Therapist introduction	37/37	100
Therapist registration	37/37	100
Therapist consent (online)	37/37	100
**Therapist questionnaires**		
Therapist SUS (month 3)	19/37	51.4
Therapist SUS (month 6)	16/37	43.2

* SUS was only performed for intervention arm participants

### Adverse events

Data on all adverse events (AE) and serious adverse events (SAE) that affected patients through their participation was collected.

In total, there were 2 AEs reported in the study, both of which occurred to participants in the intervention arm. The severities of these events were categorised as mild and moderate, neither event was deemed to have been caused by the intervention.

A total of 4 SAEs were reported, 3 in the intervention group, and 1 in the control group. Two of these events were deemed serious and required inpatient hospitalisation and a delay in study participation. None of the events were related or caused by the intervention or study procedures.

### Economic evaluation results

#### Feasibility of data collection

The Client Services Receipt Inventory (CSRI) was used to collect participant-reported health and social care service use related to upper limb rehabilitation. Data completeness was high amongst both groups, with 86.7 and 80.0% of CSRI forms returned at 6 and 12 weeks respectively. The tool was generally well-integrated into trial procedures, although some inconsistencies in reporting were noted. These included variation in participant recall, difficulty attributing services specifically to arm rehabilitation, and inconsistent reporting of out-of-pocket expenses.

Treating therapists recorded inpatient days in Hyper-Acute Stroke Units (HASU) and Acute Stroke Units (ASU), as well as outpatient sessions delivered through Early Supported Discharge (ESD) and Community Neurological Rehabilitation Teams (CNRT), using electronic spreadsheets in Microsoft Excel. These data were consistently recorded across sites, although the burden of manual data entry varied. A summary of data sources and relevant observations is shown in Table 8.

**Table 8:** Usual care cost categories

Category	Source	Notes
Inpatient care—HASU days	Therapist (EHR)	Consistently recorded across all sites Burden on manual data transfer for trial purposes noted
Inpatient care—ASU days	Therapist (EHR)	
Community rehab—ESD sessions	Therapist (EHR)	
Community rehab—CNRT sessions	Therapist (EHR)	
Primary care (e.g. GP appointments)	CSRI	Participant recall varied Difficulty attributing care directly to arm rehab Limited detail on medication or relevance to arm rehab Wide variation in interpretation; often not arm-specific
Secondary care (e.g. hospital and outpatient appointments)	CSRI	
Other interventions or treatments (e.g. splints, FES)	CSRI	
Mental health services	CSRI	
Social care services	CSRI	

### Per-patient costing of the OnTrack intervention

The total cost of delivering the intervention was £19,872, a cost of £1529 per participant. A breakdown of intervention-related costs is presented in Table 9.

**Table 9:** Costs of intervention

Cost component	Total cost	Description
Devices (watch + phone)	£3250	Hardware provided to intervention participants
Technical support	£375	Setup, maintenance, and troubleshooting
Therapist training	£15,852	Training delivered to 42 therapists
Follow-up calls	£368	Therapist calls with participants post-discharge
Dashboard consultations	£27	Therapist time reviewing participant data
**Total**	**£19,872**	

### Combined costs summary

Total costs per participant were calculated by combining intervention costs, therapist-recorded usual care resource use and participant-reported CSRI data. Because of the unequal number of participants that completed 12-week follow-up (13 in the intervention group and 11 in the control group), the costs were summarised separately using total and mean values. The combined cost summary provided a complete picture of the costs involved in the analysis. The total cost in the intervention arm was £95,399 compared to £154,709 in the control group, a difference of £59,311. The main cost drivers were inpatient care (particularly ASU, HASU, and CNRU), Table 10 presents a breakdown of combined costs across categories.

**Table 10:** Combined cost summary

Cost by category of care	Combined	Intervention	Usual care	Change
**CSRI**				
Primary care appointments	£213	£132	£82	£50
Secondary care outpatient appointments	£14,272	£7390	£6882	£508
Secondary care admissions	£291	£291	£0	£291
Secondary care tests	£1741	£176	£565	£611
Medication/equipment	£17	£15	£2	£14
Complementary therapies (e.g. splints, electrical stimulation, botox)	£17,732	£7976	£9756	− £1780
Psychological support	£6919	£1870	£5049	− £3179
Social care	£10,574	£2979	£7595	− £4616
**Therapist reported costs**				
Inpatient care (ASU, HASU, CNRU)	£160,975	£43,155	£117,820	− £74,665
Community care (ESD, CNRT)	£17,502	£10,543	£6959	£3583
**Intervention costs**				
Combined intervention costs		£19,872	£0	£19,872
**Totals**		**£95,399**	**£154,709**	− **£59,311**

### Mean costs

The mean costs and standard deviations (SD) per participant were calculated for both arms of the study by combining the average intervention costs, therapist-recorded usual care resource use and participant-reported CSRI data. The mean cost was £7338 (SD: £5471) in the intervention group and £14,064 (SD: £17,148) in the control group, giving a mean difference of £6726. The large standard deviations are indicative of the small sample size and large variations within the data used for the analysis. Table 11 presents a breakdown of mean costs across all the resource categories for both study arms.

**Table 11:** Mean costs

**Cost by category of care**	**Intervention** mean (SD)	**Usual care** mean (SD)	**Change** mean
**CSRI**			
Primary care appointments	£10 (£14)	£7 (£15)	£3
Secondary care outpatient appointments	£568 (£968)	£626 (£1480)	− £57
Secondary care admissions	£22 (£81)	£0 (£0)	£22
Secondary care tests	£90 (£238)	£51 (£170)	£39
Medication/equipment	£1 (£4)	£0 (£1)	£1
Complementary therapies (e.g. splints, electrical stimulation, botox, etc.)	£614 (£1377)	£887 (£1342)	− £273
Psychological support	£144 (£316)	£459 (£691)	− £315
Social care	£229 (£377)	£690 (£860)	− £461
**Therapist reported costs**			
Inpatient care (ASU, HASU, CNRU)	£3320 (£6068)	£10,711 (£16,415)	− £7391
Community care (ESD, CNRT)	£811 (£589)	£633 (£668)	£178
**Intervention costs**			
Per-participant intervention costs	£1529 (£30)	£0	£1529
**Totals**	**£7338 (£5471)**	**£14,064 (£17,148)**	** − £6726**

Although these findings suggest a potential reduction in mean cost, the small sample size and baseline imbalances in the number of patients in both arms make it unlikely to conclude that these reflect true differences attributable to the intervention. However, the results support the need for an expanded study to capture long-term relevant cost data for a future economic evaluation.

### Out-of-pocket costs

Some of the care received by participants was paid privately. The mean cost of privately paid care for the intervention group was £533 (SD: £1397) compared to £1083 (SD: £1625) for the usual care group, a mean cost difference of £550.

### Utility data and EQ-5D-5L

Utility data were collected using the EQ-5D-5L at baseline, 6 weeks and 12 weeks. These responses were converted into EQ-5D-3L utility values in line with the NICE reference case for economic evaluations [51]. The aim was not to estimate QALYs but to assess the feasibility and completeness of utility data collection for future modelling.

Baseline differences in utility were observed between the groups, with control participants reporting higher utility scores at baseline (mean difference − 0.0685). A marginal increase in utility was seen in the intervention arm at 6 weeks compared to control (+ 0.0012), but this was not sustained at 12 weeks (− 0.0068). These fluctuations are likely to reflect random variation given the small sample size and short follow-up period. Table 12 summarises the converted utility values from EQ-5D-5L to EQ-5D-3L at each time point.

**Table 12:** EQ-5D-3L utility data converted from EQ-5D-5L

Converted EQ-5D-5L	Intervention mean (SD)	Usual care mean (SD)	Change
Baseline	0.3597 (0.2376)	0.4282 (0.3798)	− 0.0685
Week 6	0.4865 (0.1568)	0.4853 (0.2874)	0.0012
Week 12	0.5750 (0.2238)	0.5818 (0.3029)	− 0.0068

These results suggest that whilst it is feasible to collect EQ-5D-5L data in this population, observed differences in utility scores should not be overinterpreted. In a future trial, a longer follow-up period and larger sample size would be required to produce meaningful estimates of quality-adjusted life years (QALYs) and assess the potential health benefits of the OnTrack intervention.

### Cost consequence analysis

A cost-consequence analysis (CCA) was conducted to summarise the comparative costs and outcomes across trial arms. This approach, recommended for early evaluations of digital health interventions [34], presents costs and outcomes separately to support transparent reporting.

As shown in Table 13, the intervention arm was associated with lower mean total costs per participant and a slight improvement in utility at 6 weeks, though this was not sustained. These exploratory results should be interpreted with caution, as the study was not powered to detect economic or clinical differences.

**Table 13:** Cost consequence analysis

**Costs**	**Intervention** **mean (SD)**	**Usual care** **mean (SD)**	**Change**
	£7338 (£5471)	£14,064 (£17,148)	− £6726
**Consequences** **(Converted EQ-5D-5L)**	**Intervention** **mean (SD)**	**Usual care** **mean (SD)**	**Change**
Baseline	0.3597 (0.2376)	0.4282 (0.3798)	− 0.0685
Week 6	0.4865 (0.1568)	0.4853 (0.2874)	0.0012
Week 12	0.5750 (0.2238)	0.5818 (0.3029)	− 0.0068

Taken together, this exploratory CCA demonstrated that cost and outcome data could be collected and analysed using a cost-consequence framework. These methods can be refined and scaled in a future definitive trial to support robust economic evaluation.

## Discussion

This study evaluated the feasibility of delivering the OnTrack intervention within NHS stroke services, assessing recruitment, retention, intervention adherence, and fidelity of delivery. The trial successfully recruited 30 participants across acute and community stroke services achieving 71.4% of its target sample size. Retention rates were high, with 80% of participants completing follow-up assessments at 12 weeks. Intervention adherence was excellent, with participants engaging with the OnTrack system on an average of 6.3 days per week, reflecting high acceptability and indicating the feasibility of self-directed rehabilitation. Fidelity of intervention delivery was 53.9%, highlighting areas for refinement in therapist training and intervention implementation—detailed findings on this regard were explored in full as part of an independent process evaluation.

As observed in this study and others, loss to follow-up was more common in the usual care group. This differential attrition introduces a risk of bias, as it is unclear whether participants lost to follow-up were showing the least or most improvement. In future trials, strategies should be explored to maintain engagement in the control group and reduce attrition. One potential approach could be to provide control group participants with access to selected elements of the intervention, such as self-management information or light-touch digital materials, without offering the full OnTrack programme. This would ensure participants feel supported and valued, whilst preserving the distinction between arms for analytic purposes, however, this can also pose the risk of turning the group into an active control group. The timing and delivery of such materials would need careful consideration to avoid contamination but could be introduced, for example, at randomisation or mid-way through the study to maintain interest and improve retention.

Secondary outcomes, including measures of stroke-related impairment, arm function, self-efficacy, quality of life, health resource use, and intervention usability were collected successfully, indicating feasibility of data collection for a future trial. Whilst the study was not powered to detect significant clinical differences, trends suggested improvements in different areas for both groups.

The preliminary health economic evaluation also demonstrated the feasibility of collecting cost and utility data to inform a future full-scale trial. A per-patient costing approach was used to estimate the cost of delivering the OnTrack intervention, and the CSRI was used alongside clinician-reported data to assess health and social care resource use. Although the intervention group appeared to have lower costs (− £6726), this likely reflected baseline differences and random variation in a small, underpowered sample. Utility data, converted from EQ-5D-5L to EQ-5D-3L, were largely complete but showed minimal and inconsistent differences between groups over time. Future studies would benefit from incorporating validated, stroke-specific instruments for arm impairment rehabilitation assessments (e.g. Fugl-Meyer Assessment [52–54], Action Research Arm Test [55, 56], Wolf Motor Function Test [57]) Importantly, these exploratory findings confirm the feasibility of cost data collection, whilst also highlighting areas for refinement, including improvements to data attribution, recall accuracy, and linkage with routinely collected electronic health record data. Future studies should include a longer follow-up period to assess cost-effectiveness and quality-adjusted life years (QALYs) over time.

The findings align with previous research demonstrating the challenges of increasing rehabilitation intensity post-stroke within routine clinical practice [11]. Digital interventions, such as virtual reality and robotic-assisted rehabilitation, have shown potential in enhancing arm and hand recovery [15, 16]; however, implementation of these solutions across usual care practices has remained limited due to cost and resource constraints [9]. The high adherence rates observed in this study suggest that wearable technologies, supported by personalised self-management coaching, such as OnTrack, may offer a scalable and accessible alternative to increase rehabilitation intensity beyond formal therapy sessions.

Process evaluation findings will be reported separately; as expected, these highlight the importance of therapist engagement in successful delivery of digital interventions. Insights gathered are consistent with other studies showing that therapist involvement improves patient engagement with digital rehabilitation tools [58]. On the other hand, the moderate intervention fidelity scores observed in this study indicates that further refinement of training and support strategies will be needed to optimise implementation in a future trial.

### Strengths and limitations

A key strength of this study is its pragmatic design, conducted across acute and community NHS stroke services. The structured feasibility framework and predefined progression criteria offer clear guidance for assessing readiness for a full-scale randomised controlled trial. Additionally, the inclusion of a cost consequence analysis and embedded mixed-methods process evaluation enhances the real-world relevance of the findings.

Several limitations must be acknowledged. First, whilst recruitment targets were not fully achieved, the recruitment rate was consistent with similar studies, which typically recruit one or two participants per site per month [41]. Nonetheless, the recruitment process encountered difficulties, particularly in acute hospital settings, due to patient acuity and time constraints. Future studies may benefit from expanding the inclusion window to enable anyone undergoing arm rehabilitation treatment to participate and allowing baseline assessments to be conducted across multiple sessions to reduce burden.

Although the trial aimed for participants to complete participation at 12 weeks, some follow-ups were conducted beyond this period. These delays were most commonly due to practical constraints, including participant availability and coordination of in-person or remote assessments. In a few cases, longer delays were associated with adverse events such as hospital admissions or periods of medical instability that required temporary suspension of trial activities. Whilst these events are not uncommon in post-stroke populations, they highlight the importance of building flexibility into follow-up protocols in future trials. Future studies should consider contingency plans for managing delays due to health-related interruptions, without compromising data integrity or participant safety.

Arm activity was only monitored in the intervention group, limiting direct comparisons of activity and engagement specifically with arm therapy. Future studies should consider ways to monitor arm activity in the control group, potentially via passive wearable tracking without feedback, to enable clearer understanding of differences in behavioural engagement and recovery.

Whilst the CSRI was useful for estimating health resource use, several limitations were noted. Some reported use may not have been directly related to upper limb rehabilitation, and data quality was affected by participant recall bias. Additionally, in-hospital care and discharge information obtained from clinicians required significant manual input and follow-up. Direct access to routinely collected healthcare episode data and refinement of data collection methods would likely improve efficiency and accuracy.

Finally, although a full economic evaluation was not the objective of this trial, the exploratory cost-consequence analysis suggested potential cost differences between groups. These findings must be interpreted cautiously due to the trial’s limited power, short follow-up, and possible baseline imbalances. Nonetheless, the methods and tools applied have proven feasible and can be refined for future research.

### Implications for future studies and implementation

The findings support the feasibility of delivering OnTrack within NHS stroke services and highlight key considerations for scaling up to a definitive RCT. Strategies to enhance recruitment and data collection, such as the ones described previously, should be considered. Refinement to therapist training may also improve fidelity of intervention delivery.

Beyond the context of this trial, the results demonstrate the potential for integrating digital self-management interventions into routine stroke rehabilitation pathways. The NHS review by Lord Darzi [13] emphasised the need for innovative digital solutions to address the growing demand for rehabilitation services. OnTrack aligns with these priorities by promoting increased rehabilitation intensity through self-directed activity supported by real-time feedback and behaviour coaching. Future research should evaluate the longer-term clinical and economic impact of OnTrack and its implementation across the wider health system.

## Conclusion

The UPBEAT Trial demonstrated that OnTrack is acceptable and feasible for stroke survivors and can be implemented within NHS stroke services with strong engagement and adherence rates. Whilst recruitment challenges and moderate fidelity were observed, the trial met key feasibility criteria and identified clear areas for refinement. The feasibility of collecting cost and utility data has also been demonstrated, supporting the development of a robust economic evaluation in a full-scale trial. These findings contribute to the growing evidence base for digital rehabilitation interventions and their role in enhancing stroke recovery and service delivery.

## Data Availability

Trial data is stored on secure servers maintained on the Cardiff University network. CTR is a signatory of AllTrials and aims to make its research data available wherever possible. Data requests will undergo a review process to ensure that the proposal complies with patient confidentiality, regulatory and ethical approvals and any terms and conditions associated with the data. Trial data can be made available upon reasonable request and following CTR data sharing policies. Requests to access the data should be made to ctrdatasamplerequests@cardiff.ac.uk.

## Abbreviations

MAL-14 QOM: Motor Activity Log, Quality of Movement Scale
SSEQ: Stroke Self-Efficacy Questionnaire
mRS: Modified Rankin Scale
EQ-5D-5 L: EuroQOL-5 dimension, 5-level, health-related, quality-of-life questionnaire
SUS: System Usability Scale
CSRI: Client Services Receipt Inventory
ASU: Acute Stroke Unit
CLCH: Central London Community Healthcare NHS Trust
CNRT: Community Neuro Rehabilitation Team
CNRU: Clinical Neurological Rehabilitation Unit
CSGUL: City St George’s University of London
CTR: Centre for Trials Research
HASU: Hyper Acute Stroke unit
HRCH: Hounslow and Richmond Community Healthcare NHS Trust
ICHT: Imperial College Healthcare NHS Trust
ICHP: Imperial College Health Partners
ICL: Imperial College London
NHS: National Health Service
NICE: National Institute for Health and Care Excellence
PI: Principal Investigator
PPIE: Patient and Public Involvement and Engagement
RCT: Randomised controlled trial
REC: Research Ethics Committee
RGIT: Research Governance and Integrity Team
